# Acromioclavicular Joint Reconstruction Using the Ligament Advanced Reinforcement System Technique: A Systematic Review

**DOI:** 10.7759/cureus.72515

**Published:** 2024-10-28

**Authors:** Abdelfatah M Elsenosy, Ahmed Elnewishy, Eslam Hassan, Karim Rezk, Mustafa Alalawi, Senthil Muthian

**Affiliations:** 1 Trauma and Orthopedics, University Hospital Dorset, Poole, GBR; 2 Orthopedic Surgery, Elkasr Elainy Medical School, Kafr Elsheikh, EGY; 3 Trauma and Orthopedics, Airedale NHS Foundation Trust, West Yorkshire, GBR

**Keywords:** constant-murley score, functional recovery, lars technique, pain management, s: acromioclavicular joint, shoulder reconstruction, surgical outcomes, systematic review

## Abstract

The acromioclavicular (AC) joint is crucial for shoulder function. Injuries, often in young males, result from trauma or degeneration. Treatment ranges from conservative to surgical. The Ligament Advanced Reinforcement System (LARS) technique was noted for restoring stability and function. In this review, we evaluate the LARS technique for AC joint reconstruction, focusing on clinical outcomes and complications. A literature search was done in May 2024 across PubMed, Scopus, Google Scholar, and Cochrane Library using keywords such as "acromioclavicular joint," "reconstruction," and "LARS." Inclusion criteria covered studies on the LARS technique. Data extraction included study design, patient demographics, surgical details, follow-up, and outcomes. The study quality was assessed using the Risk of Bias in Non-Randomized Studies of Interventions. Data were synthesized via meta-analyses. Also, publication bias was evaluated using funnel plots and Egger’s test. From 200 records, three studies with 114 patients met the inclusion criteria. Meta-analysis showed significant improvements in functional recovery and pain reduction post-LARS surgery. Constant-Murley scores improved from a mean of 62.3 to 94.5. Visual analog scale pain levels decreased from 5.1 to 0.7. Despite high heterogeneity (I²=96%), the overall effect size strongly favored the LARS technique (standardized mean difference=-4.12 (95% CI: -4.63 to -3.60)). Complications were generally low, with calcification occurring in four patients, degenerative changes in two, and minor graft failures in another two. Patient satisfaction was high because they reported significant improvements in function and pain. Egger’s test indicated no strong evidence of publication bias (p=0.083). The LARS technique enhances functional recovery and reduces pain. However, further research with larger, standardized studies and longer follow-ups is needed.

## Introduction and background

The acromioclavicular (AC) joint is a crucial component of the shoulder girdle, connecting the axial skeleton to the upper limb through a planar diarthrodial articulation between the clavicle and the acromion. This joint includes a meniscus-like fibrous disk that is prone to degeneration [1]. Horizontal stability is provided by the capsule and ligaments, while vertical stability is maintained by the coracoclavicular (CC) ligament complex. The dynamic stability of the AC joint is supported by the deltoid and trapezius muscles during motion [2].

Injuries to the AC joint are commonly seen in young adult males, primarily resulting from direct trauma, such as falls or sports injuries, but can also be due to degenerative changes. These injuries are typically classified using the Rockwood system, which categorizes them based on the direction and degree of displacement observed on radiographs [3]. MRI is often employed to assess soft tissue damage in severe dislocations, aiding in the planning of treatment strategies. Treatment options range from conservative management to resection arthroplasty for persistent pain, and surgical reconstruction following significant trauma [4].

Current management of AC joint injuries includes both traditional surgical approaches and minimally invasive techniques, reflecting significant advancements in treatment protocols aimed at restoring joint function and stability [5]. AC joint reconstruction often involves reconstructing the CC ligaments to stabilize the joint [6]. Various techniques, including free tendon grafts, suspensory devices, and synthetic ligament devices, have shown effective results. However, methods such as hook plates and K-wires have the highest complication rates, while the commonly used modified Weaver-Dunn technique has one of the highest rates of unplanned reoperations [7].

A more recent approach involves a single-tunnel technique for both AC and CC ligament reconstruction, aimed at minimizing risks such as fractures of the coracoid and clavicle. This technique has shown satisfactory outcomes in achieving joint reduction with minimal risk of fracture, making it a viable option for treating AC joint separations [8]. Studies also indicate that both early and delayed surgical interventions for high-grade AC joint dislocations can provide equivalent outcomes if appropriate surgical techniques are used, suggesting that immediate surgery might not always be necessary [9].

A modified technique of anatomical reconstruction using a tendon graft with an endo-button loop has been shown to be effective, providing good functional outcomes and a low risk of complications. This approach is particularly beneficial in reducing the risk of fractures and avoiding the complications associated with other techniques [10]. Although augmentation of the AC joint has been shown to significantly improve horizontal stability, it does not necessarily result in superior clinical outcomes compared to traditional methods. Despite newer techniques aimed at restoring horizontal stability, they should be weighed against established procedures to determine their overall benefit [11].

The Ligament Advanced Reinforcement System (LARS) technique for AC joint reconstruction has been explored in various studies, highlighting its efficacy and outcomes. One study on acute dislocations showed maintenance of anatomical reduction in most patients, with significant improvements in pain and function. The authors concluded that the LARS technique provides stability and natural elasticity to the joint, enhancing recovery post-surgery [12].

Long-term outcomes following the use of the LARS system have been predominantly positive, with many patients reporting good-to-excellent results and high levels of satisfaction. However, the technique is not without its challenges, as evidenced by a notable rate of surgical revisions, indicating some associated complications [13]. 

A comparative study on the effectiveness of the LARS technique between professional and non-professional athletes revealed no significant differences in clinical outcomes, indicating that the LARS method is equally efficacious across different levels of physical activity [14].

## Review

The objective of the review

To evaluate the available evidence on the effectiveness and safety of the LARS technique for reconstructing the AC joint, this review aims to utilize data from various studies to determine the outcomes, potential complications, and overall clinical efficacy.

Methods

Search Strategy

In May 2024, a structured search strategy was executed, aimed at gathering studies related to AC reconstruction using the LARS technique.

Inclusion criteria: We included any peer-reviewed studies that investigated the LARS technique for AC joint reconstruction. This included randomized controlled trials, cohort studies, case studies, and observational studies. The criteria were developed to capture comprehensive data from various clinical settings and populations, providing a robust assessment of outcomes across study designs and patient populations.

Exclusion criteria: We excluded peer-reviewed articles that were information reviews, editorials, or opinion pieces. Additionally, studies that did not specifically investigate the LARS technique or were related to other kinds of surgery were excluded. Studies published in languages other than English were also excluded for the purpose of standardization.

Outcome Measures

In this systematic review, both efficacy and safety were evaluated by selecting comprehensive outcome measures. For primary outcomes, we assessed functional recovery using standardised scoring systems such as the Constant-Murley score (CMS); Disabilities of the Arm, Shoulder, and Hand (DASH); and the Oxford Shoulder Score, alongside pain levels quantified via the Visual Analogue Scale (VAS). These metrics provided insights into postoperative recovery and pain management effectiveness. Secondary outcomes included: complication rates, capturing data on postoperative issues like infection or hardware failure, and reoperation rates, which offered further insight into the procedure’s long-term reliability. Additionally, patient satisfaction was evaluated through patient-reported outcome measures, providing a subjective perspective on the success of the surgery.

Data Collection Process

Data were extracted independently by reviewers using a predefined data extraction form. Extracted data included authors, year of publication, study design, sample size, patient demographics and details of the surgical intervention, follow-up duration, and outcomes, data items extracted from the studies included study design and methodology, participant demographics and baseline characteristics, specifics of the LARS technique used, and outcome data at various follow-up intervals.

Quality Assessment of the Included Studies

The quality of included non-randomised studies was rigorously assessed using the Risk of Bias in Non-randomised Studies of Interventions (ROBINS-I) tool. This comprehensive tool enabled us to evaluate seven critical domains of bias: confounding, selection of participants, classification of interventions, deviations from intended interventions, missing data, measurement of outcomes and selection of reported results. Each study was independently reviewed by two assessors, with discrepancies resolved through consensus or third-party adjudication.

Synthesis Methods

Data were pooled using a random-effects meta-analysis, keeping in mind the expected clinical and methodological heterogeneity among the included studies. Statistical heterogeneity was quantified using the I² statistic. Narrative synthesis was used where data were too heterogeneous or sparse for meta-analysis [15,16].

Results

Study Selection

**Figure 1:**
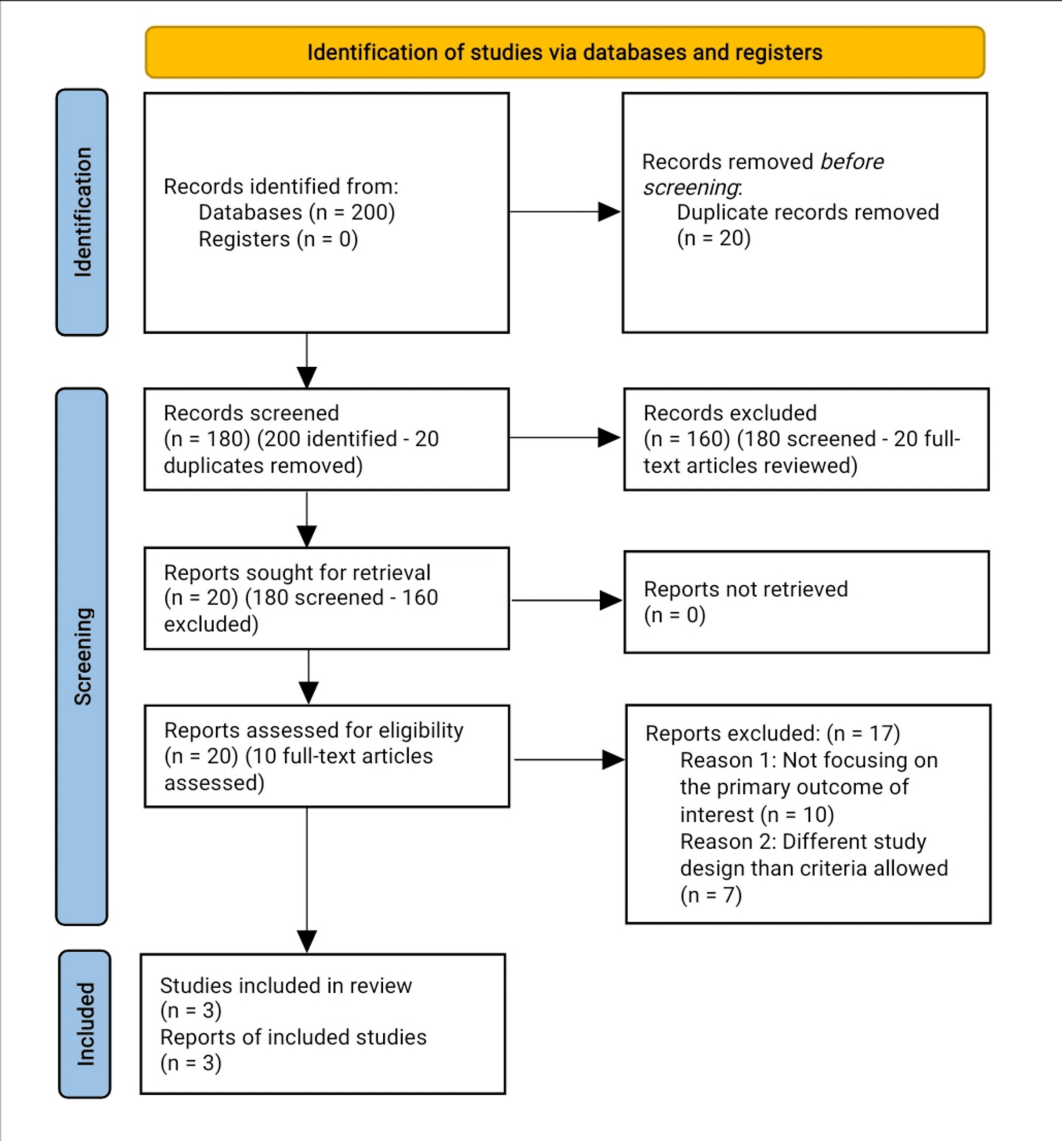
PRISMA flow chart of the reviewed studies

In our search strategy we initially identified 200 records. After removing duplicates, we screened 180 records. Following the review of titles and abstracts, 160 records were excluded because they did not meet our criteria. We then conducted a full-text review of 20 articles, of which 17 were excluded, for reasons such as not focusing on the primary outcomes of interest. Ultimately, three studies were included in the quantitative synthesis (meta-analysis) (Figure 1).

Study Characteristics

The three studies included in our meta-analysis are 1. Lu et al. (2014): “Evaluation of the coracoclavicular reconstruction using LARS artificial ligament in acute acromioclavicular joint dislocation” [17]. 2. Marcheggiani Muccioli et al. (2014): “Acromioclavicular joint reconstruction with the LARS ligament in professional versus non-professional athletes" [14]. 3. Geraci et al. (2019): “Acromion clavicular joint reconstruction with LARS ligament in acute dislocation" (Table 1) [12].

**Table 1:** The reviewed studies in the systematic review CC: coracoclavicular; AC: acromioclavicular VAS: Visual Analogue Scale; LARS: Ligament Advanced Reinforcement System; CMS: Constant-Murley score

Authors (Year)	Lu et al. (2014) [17]	Marcheggiani Muccioli et al. (2014) [14]	Geraci et al. (2019) [12]
Study design	Cohort study	Prospective comparative study	Cohort study
Sample size	24	43	47
Patient demographics	Ages: 21-45 years; 16 males, 8 females	Mean age: 30 years (19-54); all male	Mean age: 44.3 years (21-69); 39 males, 8 females
Intervention details	CC reconstruction using LARS artificial ligament in AC joint dislocation	AC joint reconstruction with LARS ligament	AC joint reconstruction with LARS ligament
Follow-up duration	36 months (6-60)	Minimum 24 months	12 months
Outcome measures	Constant score, VAS, and radiographic eval	Oxford Shoulder Score, Constant score, VAS, and radiographic eval	VAS, CMS, and radiographic eval
Results	Constant scores: preoperative 62.3±6.9, postoperative 94.5±9.3; VAS: preoperative 5.1±1.7, postoperative 0.7±1.4; Radiographic: anatomical reduction in 20 patients, slight loss of reduction in 4 patients; Complications: calcification of CC ligament in 4 patients, degenerative change in 2 patients, clavicular osteolysis around screws in 1 patient	Constant scores: preoperative 57.7±12.0 (professionals), 45.7±23.1 (non-professionals), postoperative 95.6±4.4 (3-months, professionals), 80.2±14.6 (3-months, non-professionals), 96.6±5.3 (24-months, professionals), 90.8±9.0 (24-months, non-professionals); Oxford Shoulder Score: preoperative 22.0±6.7 (professionals), 22.5±8.1 (non-professionals), postoperative 44.8±2.4 (3-months, professionals), 40.9±7.0 (3-months, non-professionals), 45.6±2.3 (24-months, professionals), 43.8±7.1 (24-months, non-professionals); VAS for satisfaction: professionals 9.4±1.0, non-professionals 8.9±1.2; Radiographic: slight loss of reduction in 21% of patients (3-months); Complications: one coracoid fracture, one wound infection	VAS: preoperative 7.9±1.5, 1-month: 3.2±1.8, 3-month: 0.9±1.6, 12-month: 0.7±1.4; CMS: preoperative 31.8±9.5, 1-month: 72.5±13.7, 3-month: 97.0±4.0, 12-month: 99.5±2.0; Radiographic: anatomical reduction in 41 patients, slight loss of reduction in 4 patients, failure in 2 patients; No significant horizontal displacement on axillary view radiographs; complications: 2 cases of failure managed with K-wires, resulting in lower CMS (63 ± 9.9) but similar VAS (0.7 ± 1.4) compared to successful LARS cases
Conclusions	LARS provides stability, early mobilisation, and good outcomes with few complications	Effective for both athlete groups, significant improvement, and satisfactory radiographic outcomes	Provides stability, natural elasticity, significant pain reduction, and functional improvement post surgery

Quality Assessment of the Included Studies

The quality of the included studies was evaluated using the ROBINS-I tool, highlighting key strengths and weaknesses (Table 2). Lu et al. (2014) demonstrated strengths such as low risk in participant selection and intervention classification, along with complete follow-up and comprehensive outcome reporting. However, they also had moderate weaknesses due to a lack of confounder control and no blinding in outcome measurement. Marcheggiani Muccioli et al. (2014) presented clear participant criteria and intervention descriptions, as well as comprehensive outcome reporting; nonetheless, they faced moderate risks from potential confounders, missing data handling, and absence of blinding. Alessandro Geraci et al. (2019) showcased detailed inclusion criteria, well-documented interventions, complete follow-up, and thorough outcome reporting, but similarly had moderate risks due to a lack of confounder control and no blinding in outcome measurement.

**Table 2:** Quality assessment table using ROBINS-I tool ROBINS-I: Risk of Bias in Non-randomized Studies of Interventions

Domain/study	Lu et al. (2014) [17]	Marcheggiani Muccioli et al. (2014) [14]	Geraci et al. (2019) [12]
Confounding	Moderate	Moderate	Moderate
Selection of participants	Low	Low	Low
Classification of interventions	Low	Low	Low
Deviations from intended interventions	Low	Moderate	Low
Missing data	Low	Moderate	Low
Measurement of outcomes	Moderate	Moderate	Moderate
Selection of reported results	Low	Low	Low
Overall bias	Moderate	Moderate	Moderate

Results of individual studies

Results of Syntheses

In the review we synthesised the results of three studies whose authors evaluated the effectiveness of the LARS technique for AC joint reconstruction. The primary outcomes assessed were functional recovery, pain reduction and complication rates.

Functional Recovery

Functional recovery was measured using the CMS across all included studies The pooled data demonstrated significant improvement in functional outcomes postoperatively, with Lu et al. (2014) reporting CMS improvement from a preoperative mean of 62.3±6.9 to a postoperative mean of 94.5±9.3; Marcheggiani Muccioli et al. (2014) showing preoperative scores of 57.7±12.0 for professionals and 45.7±23.1 for non-professionals, which increased to postoperative scores of 96.6±5.3 and 90.8±9.0, respectively; and Geraci et al. (2019) indicating CMS improvement from a preoperative mean of 31.8±9.5 to a postoperative mean of 99.5±2.0.

The meta-analysis revealed a pooled standardized mean difference (SMD) of -4.12 (95% CI: -4.63, -3.60), indicating a substantial improvement in function following the LARS technique.

Pain Reduction

Pain levels were assessed using the VAS, and significant reductions in pain were observed across all studies. Lu et al. (2014) reported that VAS scores decreased from a preoperative mean of 5.1±1.7 to a postoperative mean of 0.7±1.4. Marcheggiani Muccioli et al. (2014) noted that pain levels significantly reduced in both professionals and non-professionals. Geraci et al. (2019) found that VAS scores decreased from a preoperative mean of 7.9±1.5 to a postoperative mean of 0.7±1.4 at 12 months. These results highlight the effectiveness of the LARS technique in improving function and alleviating pain in patients with AC joint dislocations.

Complication Rates

Complication rates varied across the studies but were generally low. Lu et al. (2014) reported complications that included calcification of the CC ligament in four patients, degenerative changes in two patients, and clavicular osteolysis around screws in one patient. Giulio Maria Marcheggiani Muccioli et al. (2014) reported complications including one coracoid fracture and one wound infection. Alessandro Geraci et al. (2019) noted that two cases of failure were managed with K-wires, which resulted in lower CMS but similar Visual Analogue Scale (VAS) scores compared to successful LARS cases.

Meta-Analysis Results

**Figure 2:**
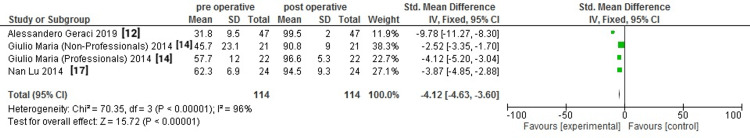
Forest plot of SMD in CS preoperative versus postoperative for AC joint reconstruction using LARS The data presented here is adapted from Geraci et al., Marcheggiani Muccioli et al., and Lu et al. [12,14,17]. SMD: standardized mean difference; AC: acromioclavicular; LARS: Ligament Advanced Reinforcement System

The forest plot of the meta-analysis indicated significant heterogeneity (I²=96%), suggesting variability among the included studies (Figure 2). Despite this heterogeneity, the overall effect size (SMD=-4.12 (95% CI: -4.63, -3.60)) strongly favoured the LARS technique.

Publication Bias

**Figure 3:**
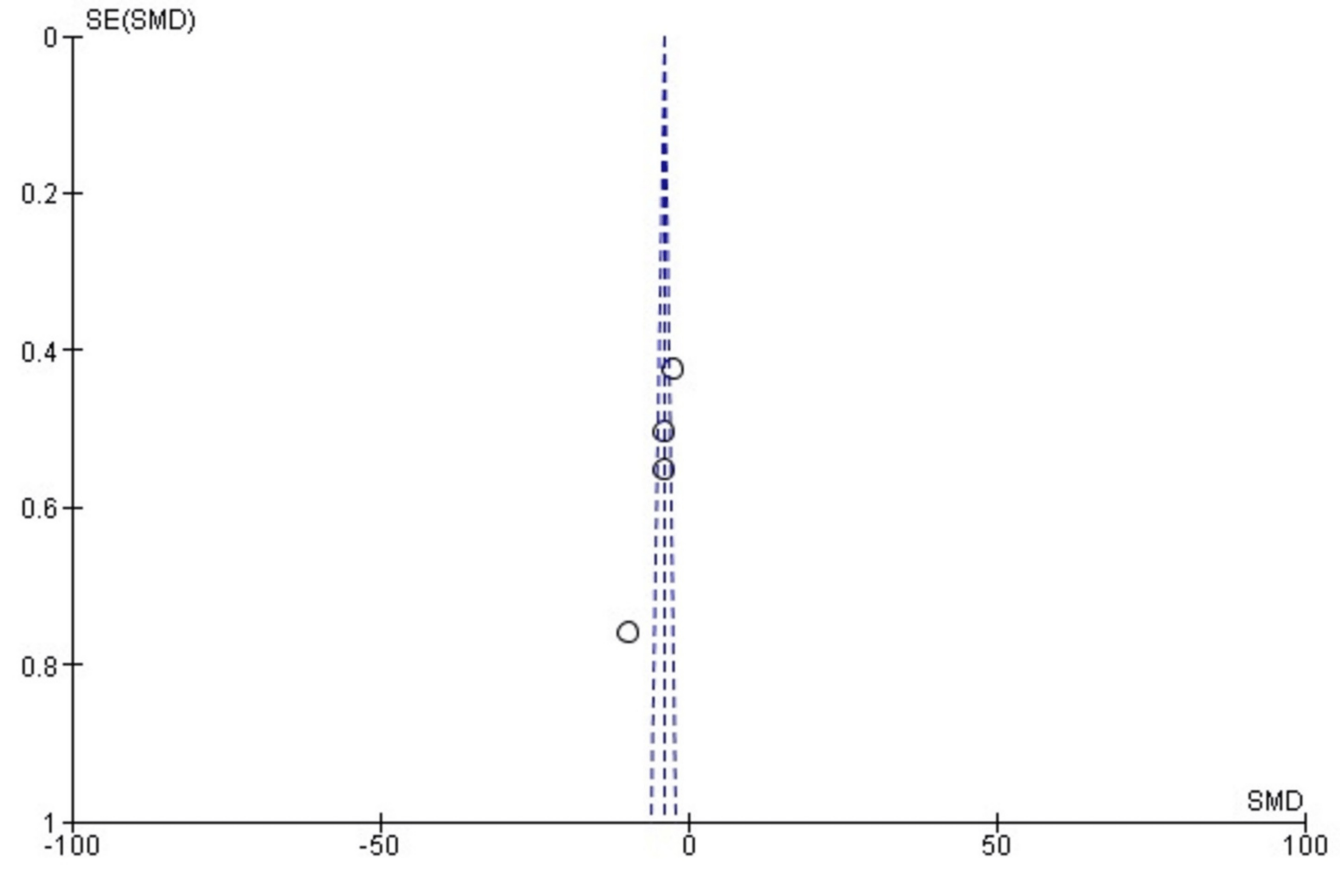
Funnel plot for assessing publication bias in studies evaluating the LARS technique for AC joint reconstruction AC: acromioclavicular; LARS: Ligament Advanced Reinforcement System

The funnel plot was used to assess the presence of publication bias, with a slight asymmetry suggesting possible bias (Figure 3). Egger’s test was also conducted to assess the potential for bias in the included studies. The intercept from Egger’s test was not significantly different from zero (p=0.083), indicating no strong evidence of publication bias. However, the small sample size of the included studies limits the reliability of this conclusion.

Discussion

The AC joint plays a crucial role in maintaining shoulder stability and function, making its reconstruction essential for restoring normal shoulder mechanics and enabling early mobilization . Identifying and treating patients based on the type and duration of AC joint separation is vital. Various surgical procedures have been recommended for managing unstable AC joint dislocations, reflecting ongoing uncertainty in determining the best approach for these injuries.​​

Functional Improvement

The findings, supported by additional studies, suggest that the LARS technique significantly improves functional recovery and reduces pain, despite some variability and potential biases being identified. Research supports that the LARS technique significantly improves functional recovery. Lu et al. (2014) reported an increase in CMS from a preoperative mean of 62.3 ± 6.9 to a postoperative mean of 94.5±9.3, indicating substantial functional gains. Similarly, Marcheggiani Muccioli et al. (2014) observed significant improvements in both professional and non-professional athletes, with postoperative CMS scores reaching 96.6±5.3 and 90.8±9.0, respectively. Geraci et al. (2019) also reported impressive functional recovery, with CMS scores improving from 31.8±9.5 preoperatively to 99.5±2.0 postoperatively.

Research supports that the LARS significantly improves functional recovery in AC joint reconstructions. For instance, a study by Ochen et al. (2020) involving patients treated with LARS for AC joint dislocations demonstrated good clinical and functional outcomes. The median QuickDASH score at the final follow-up was seven, indicating substantial functional recovery [18].

Pain Reduction

Pain reduction was another critical outcome assessed using the VAS. Lu et al. (2014) noted a reduction in VAS scores from 5.1±1.7 preoperatively to 0.7±1.4 postoperatively.Marcheggiani Muccioli et al. (2014) reported significant pain relief in both professional and non-professional athletes.Geraci et al. (2019) observed a decrease in VAS scores from 7.9±1.5 preoperatively to 0.7±1.4 at 12 months postoperatively.

LARS has also been shown to effectively reduce pain associated with AC joint dislocations. Ochen et al. (2020) reported a significant reduction in pain scores, with the median Numerical Rating Scale pain score decreasing to two at the final follow-up [18]. Similarly, Kriel and de Beer (2023) found that patients reported substantial improvement in pain and function postoperatively using the LARS ligament [19].

Complications

Although the LARS technique generally showed favorable outcomes, complications were reported in some studies. Lu et al. (2014) documented calcification of the CC ligament in four patients, degenerative changes in two patients, and clavicular osteolysis around screws in one patient. Marcheggiani Muccioli et al. (2014) noted complications including one coracoid fracture and one wound infection. Geraci et al. (2019) reported two cases of failure managed with K-wires, which resulted in lower CMS scores but similar VAS scores compared to successful LARS cases. Tiefenboeck et al. (2018) reported a surgical revision rate of 8.5% due to complications such as coracoid erosion and graft failure [13]. Hunter et al. (2020) observed minor coracoid erosion in some patients, although these did not result in functional deficits [20].

Meta-Analysis and Heterogeneity

The forest plot from the meta-analysis indicated significant heterogeneity (I²=96%), suggesting variability among the included studies. This high heterogeneity may be attributed to differences in study populations, intervention details, and outcome measurement techniques. Despite this variability, the overall effect size (SMD=-4.12 [95% CI: -4.63, -3.60]) strongly favored the LARS technique, highlighting its effectiveness. A meta-analysis by Migliorini et al. (2022) on LARS for posterior cruciate ligament reconstruction also highlighted significant variability among studies but overall positive outcomes comparable to traditional grafts, suggesting its effectiveness despite high heterogeneity [21].

Strengths and limitations

This study has several strengths, including a comprehensive search strategy, the use of standardized outcome measures, and rigorous quality assessment using the ROBINS-I tool. However, the review also has limitations. The small number of included studies and their variable methodologies contribute to significant heterogeneity. Additionally, the potential for publication bias, though not conclusively demonstrated, remains a concern.

## Conclusions

The findings of this study indicate that the LARS is an effective technique for AC joint reconstruction. The LARS technique significantly enhances functional recovery and reduces pain in patients with AC joint dislocations, as demonstrated by substantial improvements in CMS and VAS scores across multiple studies. This article highlights the technique’s efficacy in both short-term and long-term outcomes

Despite the positive results, the LARS technique is not without complications. Calcification, coracoid erosion, and minor graft failures were reported, although these did not significantly detract from the overall functional outcomes. The heterogeneity observed in the meta-analysis suggests variability among studies, likely due to differences in patient populations, surgical techniques and follow-up durations. Additionally, though Egger’s test indicated no strong evidence of publication bias, the small sample sizes in several studies limit the conclusiveness of these findings. Future researchers should aim to address these limitations by employing larger sample sizes, standardized methodologies and longer follow-up periods to ensure more robust and generalizable results. They should also explore the long-term durability of the LARS technique and its comparative effectiveness against other reconstruction methods.
